# Switching cell fate by the actin–auxin oscillator in *Taxus*: cellular aspects of plant cell fermentation

**DOI:** 10.1007/s00299-022-02928-0

**Published:** 2022-10-10

**Authors:** Christina Manz, Manish L. Raorane, Jan Maisch, Peter Nick

**Affiliations:** 1 Botanical Institute, Karlsruhe Institute of Technology, Fritz-Haber-Weg 4, 76131 Karlsruhe, Germany; 2 Institute of Pharmacy, Martin-Luther-University, Halle-Wittenberg, Hoher Weg 8, 06120 Halle (Saale), Germany

**Keywords:** *Taxus*, Paclitaxel, Plant cell fermentation, Cell differentiation, Actin, Auxin

## Abstract

**Key message:**

Paclitaxel synthesis in Taxus cells correlates with a cell-fate switch that leads to vacuoles of a glossy appearance and vermiform mitochondria. This switch depends on actin and apoplastic respiratory burst.

**Abstract:**

Plant cell fermentation, the production of valuable products in plant cell culture, has great potential as sustainable alternative to the exploitation of natural resources for compounds of pharmaceutical interest. However, the success of this approach has remained limited, because the cellular aspects of metabolic competence are mostly unknown. The production of the anti-cancer alkaloid Paclitaxel has been, so far, the most successful case for this approach. In the current work, we map cellular aspects of alkaloid synthesis in cells of *Taxus chinensis* using a combination of live-cell imaging, quantitative physiology, and metabolite analysis. We show evidence that metabolic potency correlates with a differentiation event giving rise to cells with large vacuoles with a tonoplast that is of a glossy appearance, agglomerations of lipophilic compounds, and multivesicular bodies that fuse with the plasma membrane. Cellular features of these glossy cells are bundled actin, more numerous peroxisomes, and vermiform mitochondria. The incidence of glossy cells can be increased by aluminium ions, and this increase is significantly reduced by the actin inhibitor Latrunculin B, and by diphenylene iodonium, a specific inhibitor of the NADPH oxidase Respiratory burst oxidase Homologue (RboH). It is also reduced by the artificial auxin Picloram. This cellular fingerprint matches the implications of a model, where the differentiation into the glossy cell type is regulated by the actin–auxin oscillator that in plant cells acts as dynamic switch between growth and defence.

**Supplementary Information:**

The online version contains supplementary material available at 10.1007/s00299-022-02928-0.

## Introduction

According to the seminal definition by Kössel (1891), primary metabolites are required for any cell of an organism, while secondary metabolites accumulate only in specific cells and, thus, do not fulfil housekeeping functions. Still, they are relevant for survival, for instance, by supporting adaption to environmental challenges, or by steering the interaction with other organisms. In consequence, plants as sessile organisms are endowed with a particularly rich secondary metabolism with > 100,000 specific metabolites identified already (Verpoorte 1998). Many of them possess interesting biological activities and find applications, such as pharmaceuticals, insecticides, dyes, flavours, and fragrances (Goossens et al. 2003). Of particular importance is the use of plant secondary metabolites in medicine. Some of the most significant examples are morphine and related alkaloids from *Papaver somniferum* (Morimoto et al. 2001), the sesquiterpenoid artemisinin against malaria from *Artemisia annua* (Covello 2008), or the anticancer drugs vinblastine and vincristine from *Catharanthus roseus* (van der Heijden et al. 2004), or paclitaxel from the bark of *Taxus brevifolia* and other *Taxus* species (Altmann and Gertsch 2007). Globally, well over 50,000 plant species used for medicinal purposes (Gómez-Galera et al. 2007), have remained relevant. For instance, still over 60% of anticancer drugs and 75% of drugs for infectious disease are either natural products or analogues of natural products (Newman et al. 2003).

Among these plant-derived products, the tubulin-binding diterpene alkaloid taxol (paclitaxel), first isolated from the Pacific Yew (*Taxus brevifolia*), has attracted considerable attention for its efficacy against ovarian and breast cancer (for review see Malik et al. 2011). There are different related taxanes accumulating in different species of the genus (for review see Baloglu and Kingston 1999), such as 10-deacetylbaccatin III in the European Yew *T. baccata*, whose semi-synthetic derivative is commercialized under the name taxotere (docetaxel) or baccatin VI, which has also been isolated from *T. baccata* (Küpeli et al. 2003). There are analogues of paclitaxel which have proven bioactivities or can be converted to derivatives with activity comparable to paclitaxel. One example among others is baccatin VI (Kingston et al. 1999; Küpeli et al. 2003). Baccatin III is a common precursor for production of both paclitaxel and Baccatin VI. The mode of action of these taxanes is to bind to polymerised microtubules and suppressing their innate dynamics, disrupting the formation of the mitotic spindle and, thus, inducing mitotic catastrophe culminating in apoptotic cell death (for review see Jordan and Wilson 2004).

The main problem of extracting anticancer drugs from the natural sources is the low concentration of these compounds in the plant tissue. In case of *Taxus* 10,000 kg of bark are required to obtain 1 kg of paclitaxel (Vidensek et al. 1990). Another problem is the very slow growth of the trees (Vidensek et al. 1990) and the fact that the production of paclitaxel in plants is subject to seasonal fluctuations in which temperature and light play a role (Nasiri et al. 2016). In addition, extraction from the tree requires a complex system and specific purification techniques using advanced and expensive technology (Malik et al. 2011). Overharvesting has shifted individual species of Taxus, such as *Taxus wallichiana* to the verge of extinction (Farjon and Page 1999), stimulating the search for alternative strategies to harvesting directly from natural populations. Although total chemical synthesis of paclitaxel is possible in principle (Nicolaou et al. 1994), it is not economically feasible because of the complex structure of paclitaxel and the large number of steps required (Cusido et al. 2014; Lin et al. 2018). Likewise, a strategy for semi-synthesis turned out to be difficult, because protective groups had to be added and later removed, rendering the procedure laborious and hardly practical (Lin et al. 2018). Only recently, the need for protective groups has been circumvented by using the precursor baccatin III (which by itself is already a very complex molecule) to yield a bioactive derivative of paclitaxel (Thornburg et al. 2017). Metabolic engineering of heterologous systems has been employed as well. For instance, expressing taxadiene synthase allows to re-route the carotenoid precursor geranyl–geranyl pyrophosphate to yield the paclitaxel precursor taxadiene, which has been successful in tomato (Kovacs et al. 2007), and, using a His-tagged version of taxadiene synthase, in *Arabidopsis thaliana* (Besumbes et al. 2004). However, taxadiene is just the first committed step of paclitaxel synthesis.

One of the economically successful and sustainable approach to produce paclitaxel has been plant cell fermentation. These have numerous advantages over the natural plant system–independence of seasonal, rapid growth, and the possibility for standardised production meeting the criterion of Good Manufacturing Practice (for reviews see Rao and Ravishankar 2002; Yue et al. 2016). Successful scale-up to large bioreactors with 75,000 L capacity allows to produce paclitaxel efficiently and in a sustainable manner from cell cultures which allows the market leader Phyton Biotech to produce hundreds of kilograms of pure paclitaxel per year (https://phytonbiotech.com/apis/paclitaxel).

Secondary metabolites often accumulate in the context of a plant response to a particular environmental condition, and often in cells that differentiate. A classic example is the accumulation of anthocyanin in response to activation of the photoreceptor phytochrome that requires vacuolar expansion to initiate (Steinitz and Bergfeld 1977). These conditions for the activation of secondary metabolism are often not met in cell cultures, leading to very low yields for the compounds of interest. To overcome this limitation, the accumulation of secondary metabolite can be stimulated by treatments with so called elicitors, such as activators of pathogen defence or wound responses, mimicking attacks by pathogens or herbivores as conditions, where secondary metabolism is activated in the natural context. The term elicitor is here used in a different and much broader sense than in phytopathology, where elicitors designate microbial molecules activating a defence response (for review see Boller and Felix 2009). In the context of plant cell fermentation, elicitors can be either elicitors in sensu stricto, i.e., peptides or oligosaccharides from bacteria or fungi that activate plant defence, but they can also be of phytohormonal nature, such as ethylene or jasmonic acid, or they can be even abiotic, such as salts, metals, or physical factors (reviewed in Ramirez-Estrada et al. 2016). We will, in the context of the current work, use the term elicitor in this broad, biotechnological sense. One of the most potent elicitors is methyl jasmonate (MeJA), the methylated derivative of jasmonic acid that in plants acts as systemic signal for defence responses and can even convey alarm signals to neighbouring plants (Farmer and Ryan 1990). Indeed, tobacco BY-2 cells that otherwise are unable to form alkaloids can be triggered to accumulate nicotine alkaloids by elicitation with MeJA (Shoji and Hashimoto 2008; Rajabi et al. 2017). For suspension cells of *Taxus* as well, MeJA was shown to activate Geranyl–Geranyl–Pyrophosphate Synthase (*T. baccata* Laskaris et al. 1999), and the jasmonate analogue coronatine was able to induce the first committed metabolite, taxene (*T. x media*, *T. globosa*, Ramirez-Estrada et al. 2015).

On the cellular level, jasmonate signalling is often followed by a re-organisation of intracellular architecture. For instance, microtubules are specifically eliminated in response to MeJA when cells pass through S-phase, while cortical and mitotic microtubule arrays persist (Abe et al. 1990). In contrast, cortical microtubules respond to jasmonic acid by bundling as shown by live-cell imaging in grapevine cells transformed with a GFP-tagged tubulin marker (Guan et al.^2015^). The actin cytoskeleton is responsive as well. For instance, treatment of pollen tubes with MeJA leads to actin bundling, which causes a breakdown of polarity, such that bipolar pollen tubes result (Çetinbaş-Genç and Vardar 2020).

Remodelling of actin filaments often heralds ensuing terminal differentiation–a phenomenon, well known from animal cells (for review, see Gourlay and Ayscough 2005), but also observed in plant cells (for reviews, see Franklin-Tong and Gourlay 2008; Smertenko and Franklin-Tong 2008). In plant cells, the dynamic actin network subtending the cell membrane is involved in sensing of membrane integrity through superoxide generated by the plasma-membrane located NADPH oxidase Respiratory burst oxidase Homologue and responds to entry of superoxide into the cortical plasma by rapid bundling (Chang et al. 2015; Eggenberger et al. 2017). This bundling can be mitigated by auxins that can suppress actin bundling. Since superoxide is also recruited for auxin signalling, a system of two interconnected oscillators results that can switch between cell growth and cell death. How actin bundling leads to programmed cell death, is neither clear, nor is the connection inevitable, since there exist triggers that can induce actin bundling without subsequent cell death, as found recently for glycyrrhizin, the active compound of Gan Cao (*Glycyrrhiza uralensis*), a plant drug used in Traditional Chinese Medicine (Wang et al. 2021).

"The current study was motivated by the question: why has plant cell fermentation, a strategy with great potential, been successfully employed only in a limited number of cases?" One of the limitations might be that most cell cultures are not a homogenous “biomass” to use a term which is very common in bioengineering but consist of different cell types with different metabolic potencies. This heterogeneity is often perceived as a factor introducing noise into the system, interfering with standardisation and, thus, limiting the efficiency of the entire approach. However, when we consider that metabolic potencies often develop in the context of cellular differentiation (with elicitors often acting as promoter of this process), we ask the question, whether heterogeneity in these dedifferentiated cell cultures might be useful to successfully pursue plant cell fermentation. Supporting this analogy, suspension cultures of cambial meristematic cells had also been shown previously to successfully produce paclitaxel (Lee et al. 2010). Using suspension cells of *Taxus chinensis* as successful paradigm for plant cell fermentation, we investigate cellular aspects of paclitaxel synthesis. In fact, we find that the metabolic competence for product synthesis correlates with a specific cell type, which we term glossy cells. These cells are highly vacuolated and secrete lipophilic compounds (most likely paclitaxel) through multivesicular bodies. The differentiation of glossy cells seems to be under control of both, the actin cytoskeleton, and the membrane-located NADPH oxidase Respiratory burst oxidase Homologue.

## Materials and methods

### Cell cultures and subcultivation

Suspension cells of *Taxus chinensis* (Pilg.) Rehder were provided by Phyton Biotech GmbH (Ahrensburg, Germany) and were subcultivated weekly, by inoculating 1.5 g fresh weight of cells (at day 7 of the culture cycle–stationary cells) into 50 ml of fresh Gamborg B5 medium (Duchefa, Haarlem, The Netherlands) supplemented with maltose monohydrate (10 g/l), picloram (2.42 mg/l) and TDZ (0.022 mg/l). The pH of the liquid medium was adjusted to 5.6 prior to autoclaving. The cells were incubated at 23 °C under constant shaking (120 rpm) on a Unimax 2010 platform shaker (Heidolph Instruments GmbH & Co. KG, Schwabach, Germany) in 250 ml polycarbonate Erlenmeyer flasks with filter caps (Corning GmbH, Kaiserslautern, Germany) for maintenance. Experiments involving elicitation were conducted in half of the volume (0.75 g of cells in 25 ml of medium in 125 ml polycarbonate Erlenmeyer flasks with filter caps.

### Quantification of culture growth

While this is principally correct, one needs to consider that there exists no single method that ideally can fulfil this task, each approach has its limitations. This is exactly the reason, why we used several methods in parallel. To quantify culture growth, we used three approaches in parallel. As first approach, cell density was estimated by a haemocytometer (Fuchs-Rosenthal) under bright field illumination to determine the number of cells in suspension and the average length of the cell cycle. The latter was implemented using an exponential model for proliferation (N_t_ = N_0_ × e^kt^ with N_t_ = cell density at time point t, N_0_ = cell density at inoculation, *e* = Euler constant, and *k* = time constant). Data represent the mean from five biological replications.

As second method, we determine Packed Cell Volume (PCV) modified from Jovanović et al. (2010) for BY-2 cell cultures. At the sampling time, the cell suspension was vigorously mixed to ensure homogeneity, and two aliquots of 10 ml were poured into two graded 15-ml tubes (Falcon, Carl Roth, Karlsruhe, Germany). The tubes were kept upright in the fridge for three days to allow complete sedimentation of the cells. Then, the volume of the sedimented cells was read out in technical duplicates using the grading of the tube and scored as percentage of the measuring volumes (10 ml).

Now, PCV just by itself is not a very efficient readout in case of cells which do not keep constant volume throughout the culture period. Plant cells especially continue to rapidly grow in volume at the stationary phase by vacuolation. Hence, as a third approach, we measured fresh and dry weight at a given time point. To obtain a readout for fresh weight, the medium was removed from the cell suspension by vacuum filtration (Vacuum pump ME 4 NT, Vacuubrand GmbH & Co KG, Wertheim, Germany) and weighed directly. To measure dry weight, this cell material was kept at 60 °C in a drying oven over three days to and then weighed. To characterise culture growth, we also determined a growth index over 7 days (GI_7_). This was the ratio of fresh weight of the culture at day 7 (i.e., the time of subculturing) over the inoculum (1.5 g) originally added to the medium at day 0.

To follow culture growth in a non-invasive manner, we monitored the sugar consumption by means of a portable Brix refractometer (Model PAL, Atago Co. Ltd., Tokyo, Japan). Small volumes (20 µl) of vacuum-filtered medium were applied on to the refractometer to determine the refractive index by measuring the rotation of polarised light due to sugar chirality. The inferred concentration drops over the culture cycle depending on the metabolic activity of the cells. Specifically, on sugar consumption, we have found in numerous cell lines that the sharp decline of sucrose content coincides with cell expansion, probably due to the fact that cellulose is built. This decline is proportional with the number of cells and with the intensity of their expansion. Therefore, the extent of sucrose consumption can be used as proxy to monitor overall cell growth (comparable to PCV and the cell counting that was done in parallel).

### Phenotyping of *Taxus* cells

The cells were quantitatively phenotyped with respect to cellular morphology, growth patterns, cell growth, and sugar consumption. Since *Taxus* cells form aggregates hampering microscopical analyses, for some measurements, the cells were individualised by a mild treatment with 0.25% of driselase (Sigma-Aldrich, Munich, Germany) for 20 min at 23 °C. To validate the degree of standardisation of this cell line, we followed over 6 months on a weekly base the GI_7_. To quantify cell size, aliquots were sampled under sterile conditions from individual flasks over time and individual cells were captured using an AxioImager Z1 microscope (Zeiss, Jena, Germany) and the MosaiX-module sampling system (Zeiss, Jena, Germany), covering an area of approximately 2.5 mm^2^ composed of 25 individual images. Cell length *L* and width *W* were measured from the central section of the cell using the AxioVision software (Rel. 4.8.2) (Zeiss, Jena, Germany) according to Maisch and Nick (2007). From those measurements, the ratio of the long over the short axis of each cell (so called aspect ratio) was calculated as *W*/*L*. Likewise, we estimated cell volume by approximating the cell as cylinder using the formula:

$$V=\pi \times {\left(\frac{W}{2}\right)}^{2}\times L.$$

Each data point represents a population of 1000–1200 individual cells. Viability was quantified using 0.1% of a 5 mg/ml fluorescein diacetate (FDA; Merck Chemicals GmbH, Darmstadt, Germany) stock solution according to Widholm (1972). FDA is a widely used viability test with decades of experience in plant cell culture. There is substantial evidence in the scientific literature to provide sufficient proof about the use of Fluorescein diacetate as a vital stain to assay viability. The non-fluorescent FDA is membrane permeable and cleaved by cytoplasmic esterases into the fluorescent fluorescein. Since dead cells lack this enzyme activity, they will not yield a signal. The viable, green-fluorescent cells were viewed by an AxioImager Z.1 microscope (Zeiss, Jena, Germany), using the filter set 38 HE (excitation: 470 nm, beamsplitter: 495 nm and emission: 525 nm, Zeiss).

### Mitochondrial phenotyping

Mitochondria were visualised in vivo by staining with MitoTracker Red FM (Thermo Fisher Scientific Inc., Waltham, MA, USA), a far red-fluorescent dye that stains mitochondria depending on the membrane potential, unlike MitoTracker Green FM, which is independent of membrane potential (Monteiro et al., 2020). The dye was added to the cell suspension culture at a final concentration of 100 nM freshly prepared from a 100 µM stock solution in dimethyl sulfoxide (DMSO), and the cells were observed immediately without incubation or washing. As to acquire high-resolution images, an AxioObserver Z1 inverted microscope (Zeiss, Jena, Germany) was used, equipped with a laser dual spinning disc device from Yokogawa (Yokogawa CSU-X1 Spinning Disc Unit, Yokogawa Electric Dorporation, Tokyo, Japan) and a cooled digital CCD camera (AxioCam MRm; Zeiss). Pictures were recorded using the 561 nm emission line of the Ar-Kr laser and a Plan-Apochromat 63x/1.44 DIC oil objective operated via the Zen 2012 (Blue edition, Zeiss) software. To quantify the mitochondria, their coverage over the cross area of the cell was determined using quantitative image analysis (ImageJ, https://imagej.nih.gov/ij/) from confocal sections through the cell centre. After transformation into a binary image, microtubules were thresholded, and the image then inverted. Using the Analyse Particle tool (full circularity to include also interconnected mitochondria, and an area of 10-infinity square pixels), the cross-area of mitochondria over the entire cross section of the cell was determined. Each data point represents a sample of 20–80 individual cells.

### Visualisation of paclitaxel by immunofluorescence and by Nile Red

*Taxus* cells sampled from the growth phase and production phase were stained for paclitaxel by indirect immunofluorescence according to Naill and Roberts (2005). After fixation with 1% (w/v) paraformaldehyde in 10 mM phosphate-buffered saline, the cells were processed as described in Nick et al. (2000), using a mouse monoclonal anti-taxol (Santa Cruz Biotechnology, Inc., Dallas, USA) as primary, and a TRITC-conjugated goat polyclonal anti-mouse (Sigma-Aldrich, Munich, Germany) as secondary antibody. As alternative approach, we detected lipophilic compounds in samples from specified stages with 1 µg/ml Nile red (Carl Roth, Karlsruhe, Germany) diluted from a stock solution in acetone after incubation for 1 h. In both approaches, the samples were viewed by spinning-disc confocal microscopy as described above for mitochondrial analysis.

### Elicitor treatment and precursor feeding

To get insight into the signals that modulated the incidence of glossy cells and the accumulation of alkaloids, we used various elicitors. To probe for the role of basal defence, we used 100 µM of methyl jasmonate (Duchefa, Haarlem, The Netherlands) at a final concentration of the solvent ethanol of 0.02%. A potential role of hypersensitive reactions was assessed by 100 µM of salicylic acid (Sigma-Aldrich, Deisenhofen, Germany) at a final concentration of the solvent ethanol of 0.04%. To target membrane-associated actin filaments, we used aluminium chloride (Merck, Darmstadt, Germany) as aqueous solutions in 10, 50, and 100 µM. To eliminate ethylene, we administered silver nitrate (Carl Roth, Karlsruhe, Germany) in 50 or 100 µM, again as aqueous solutions. To block the membrane-located NADPH oxidase Respiratory burst oxidase Homologue, we used 20 µM of diphenylene iodonium (Sigma-Aldrich, Deisenhofen, Germany) in 0.2% DMSO. To activate auxin signalling, we used 10 µM of Picloram (Duchefa, Haarlem, The Netherlands). Appropriate controls using the final concentration of the respective solvent alone were included throughout. The concentrations for different elicitors were not chosen arbitrarily or in ignorance of physiology but based on preparatory experiments in this and many other cell lines (Eggenberger et al. 2017, Wang et al. 2022; Duan et al. 2016). The suspension culture of our Taxus cell lines form aggregates from a few to several hundred cells. Thus, these cells are very compact, and the effective concentration is much lower than the concentration added to the medium.

### Quantification of baccatin III and VI

The filtered cell material was lyophilised and extracted by a Soxhlet extractor in fifty cycles with methanol. After reducing the methanol volume by rotary evaporation, the samples were analysed by HPLC according to Witherup et al. (1989) with minor modifications as described by Bringi et al. (2012).

### Quantification of gene expression

For sampling of the cell suspension cultures, the medium was removed by a vacuum pump and approximately 100 mg of cells were transferred to 2-ml reaction tubes, shock-frozen in liquid nitrogen and kept at − 80 °C until further processing. The frozen cells were homogenised into a powder with mortar and pestle using quartz sand. The RNA was extracted using a column-based commercial protocol (innuPREP Plant RNA Kit, Analytik Jena, Jena, Germany) according to the manufacturer instructions. Potential contaminations by genomic DNA were removed by digestion with RNAse-free DNAse (Qiagen, Hilden, Germany) for 15 min at 37 °C. Quality and integrity of the extracted RNA were verified via spectrophotometry by NanoDrop (peqlab ND-1000, Erlangen, Germany) and agarose gel electrophoresis. A template of 2 μg of RNA was reversely transcribed into cDNA with the M-MuLV Reverse Transcriptase (New England Biolabs, Frankfurt, Germany). Steady-state transcript levels for Taxadiene Synthase (TS), Taxadiene 5 Hydroxylase (T5H), 10-Deacetyl-Baccatin III-10-O-Acetyltransferase (DBAT), and 3 ‘-N-Debenzoyl-2-Deoxytaxol-N-Benzoyltransferase (DBTNBT) were determined by quantitative Real-Time PCR (qRT-PCR) as described in Svyatyna et al. (2014). An initial denaturation for 3 min at 95 °C was followed by 40 cycles of denaturation for 20 s at 95 °C, annealing for 30 s at 52–58 °C depending on the transcript, and synthesis for 40 s at 70 °C. The homogeneity of the amplicons was pre-tested by semiquantitative PCR and gel electrophoresis and verified by recording a melting curve from 50 °C to 95 °C in 0.5 °C increments. The details of the oligonucleotide primers are listed in Suppl. Table S1. Steady-state transcript levels were calculated according to Livak and Schmittgen (2001) and normalised against 18S RNA as reference. Data represent mean and standard errors from three independent experiments, each in technical triplicates.

### Statistical analysis of data

Differences between treatments were tested pairwise for significance using a by two-tailed Student ‘s *t* test assuming with a confidence interval of 95% using Microsoft Excel. Comparisons of several samples were conducted using ANOVA followed by a Tukey HSD post hoc test with R version 3.5.0 assuming independence and normal distribution of values, and a confidence interval of 95%.

## Results

### Suspension cells of *Taxus* can differentiate during maturity

**Fig. 1:**
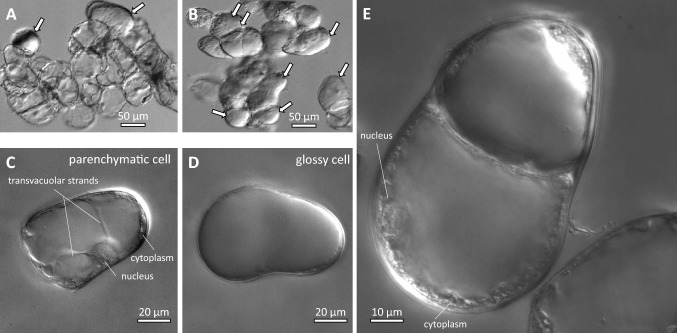
Morphological features of cell types in the suspension culture of *Taxus brevifolia* as recorded by Differential Interference Contrast. Survey of cell clusters in the initial (**A**) and progressive (**B**) state of glossy-cell formation. Glossy cells are indicated by white arrows. **C, D** Cellular details of the parenchymatic progenitor cell (**C**) and a fully differentiated glossy cell (**D**). **E** Asymmetric differentiation in a cell file, where the basal cell still partially retains feature of a parenchymatic progenitor cell

In suspension culture, the *Taxus* cells form aggregates from a few to several hundred cells. Most cells display the typical morphology of parenchymatic cells with a nucleus located in a lateral cytoplasmic pocket that is connected by transvacuolar cytoplasmic strands with the opposite flank of the cell (Fig. 1C). In the Differential Interference Contrast, the cytoplasmic strands are distinct against the translucent central vacuole. However, a minority of the cells look clearly different (Fig. 1D). They lack transvacuolar strands, and the central vacuole is reflecting the light, such that cytoplasm and nucleus are very hard to discern. Due to its bright aspect, this prominent cell type is in the following termed as “glossy cells”. Parenchymatic and glossy cells coexist in the same aggregate (Fig. 1A, B). Sometimes, asymmetric divisions can be observed, where a basal cell still maintains features of the parenchymatic state, while the apical cell already has fully expressed the glossy phenotype (Fig. 1E).

The cellular parameters of the suspension underwent characteristic dynamics that were monitored on a quantitative level (Suppl. Fig. S1). However, since the cells were growing in agglomerations, it was difficult to sample reliable data from individual cells. So, we chose a strategy, where quantification was integrating over the entire cell population. Here, the behaviour of the cell line was quite stable. When we followed the growth index (ratio of fresh weights at the end of the cultivation cycle over the initial inoculum) over half a year, it was stable around 2.5 through 15 cycles (Suppl. Fig. S1A) giving evidence for a high level of standardisation. After an initial lag of around 2 days following subcultivation, dry weight increased at a constant rate, till reaching a plateau at day 12 after subcultivation (Suppl. Fig. S1B). Fresh weight followed at a slower pace, but increased steeply after day ten after subcultivation, i.e., at a time, when the increase in dry weight had already slowed down. This indicates that during the later stage of the cycle, growth is mainly due to an increase of cell volume, while cell proliferation is contributing less to the increase in fresh weight during this time. This conclusion was supported by the time course of Packed Cell Volume (PCV), which was increasing at a slow rate in the first half of the cultivation cycle but increased steeply from day 10 (Suppl. Fig. S1C). Since cell expansion requires an elevated activity of phospholipid and cellulose synthesis for the expanding cell wall, we estimated sugar consumption from the cultivation medium, which can be done in a non-invasive manner by refractometry. The readout, as °Brix, reflects the sugar content remaining in the medium. In fact, we observed that the estimated sugar content first dropped at a slower pace, but decreased rapidly from day 10, i.e., at the time, when fresh weight (Suppl. Fig. S1B) and PCV (Suppl. Fig. S1C) increased vigorously. This quantitative phenotyping of the cultivation cycle is consistent with a pattern, where cells preferentially proliferate during the first half of the cycle, but preferentially expand during the late phase of the cycle.

### Incidence of glossy cells correlates with alkaloid accumulation

**Fig. 2:**
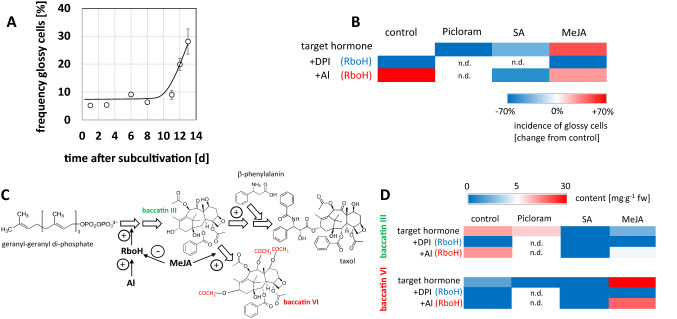
Incidence of glossy cells and link with alkaloid accumulation. **A** Frequency of glossy cells over time. Each data point represents at least 300 individual cells from three independent experimental series. **B** Factors modulating the incidence of glossy cells probed at day 6 after the respective treatment. The relative change in incidence compared to the control is indicated by a colour table. The artificial auxin Picloram was administered in a concentration of 10 µM, and the phytohormones Salicylic Acid (SA) and Methyl Jasmonate (MeJA) at 100 µM. The role of the membrane-localised NADPH oxidase Respiratory burst oxidase Homologue (RboH) was assessed either by inhibition through 20 µM diphenylene iodonium (DPI) or activation by 100 µM of aluminium chloride (Al). Data were collected from three independent experimental series, scoring between 1100 and 2300 individual cells per data point. **C** Position of the taxol precursor baccatin III and the shunt product baccatin VI in the taxol pathway and working hypothesis on their regulation. **D** Content of baccatin III and baccatin VI in response to factors that modulate the incidence of glossy cells

To get insight into the conditions that foster the appearance of glossy cells, we conducted a time course study and observed that glossy cells appear during the later stage of the cultivation cycle (Fig. 2A), from day 10, i.e., during the phase, when proliferation has slowed down, and cells undergo expansion (Suppl. Fig. S1). In the next step, we searched for conditions that would accelerate this phenomenon, scoring at day 6, when the frequency of glossy cells is usually low (Fig. 2B). In fact, we could show that addition of aluminium ions strongly promoted the formation of glossy cells, while inhibition of the NADPH oxidase Respiratory burst oxidase Homologue by Diphenylene Iodonium (DPI) was suppressive. Addition of the artificial auxin Picloram was suppressive as well, a similar, somewhat milder suppression was seen for Salicyclic Acid (SA). In contrast, Methyl Jasmonate (MeJA) was clearly inducing, and this induction could be eliminated by DPI. The effect of MeJA and aluminium was not additive, but MeJA partially mitigated the induction of glossy cells by aluminium. To probe for a link between the induction of glossy cells and the accumulation of alkaloids, we measured the levels of baccatin III, a precursor of the paclitaxel branch of the pathway, and baccatin VI, a shunt derivative of baccatin III (Fig. 2C). Here, we could observe that baccatin III was induced by aluminium ions and that this induction was completely suppressed by DPI (Fig. 2D). Furthermore, we saw an induction of baccatin VI in response to MeJA, which was mildly mitigated by simultaneous addition of aluminium ions. Thus, the pattern seen for the incidence of glossy cells is clearly reflected in the abundance of these alkaloids. The speciation into the main branch (baccatin III) and the shunt pathway (baccatin VI) was modulated by the type of elicitor, though. While both, aluminium ions and MeJA, promoted the formation of glossy cells (Fig. 2B), baccatin III, but not baccatin VI was induced by aluminium ions, while, inversely, MeJA was inducing baccatin VI, but not baccatin III. Thus, while the incidence of glossy cells correlates well with the accumulation of alkaloids, there must be a second layer deciding on the speciation of the pathway.

### Paclitaxel leaves the cells through the multivesicular body

**Fig. 3:**
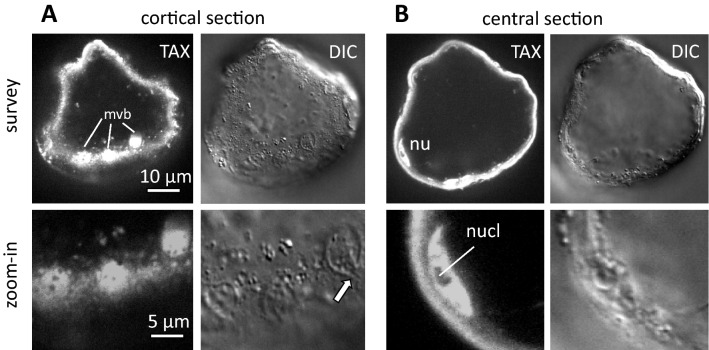
Detection of taxol by immunofluorescence in a glossy cell. Two confocal sections collected in the cortical (**A**) and the central (**B**) regions. *mvb* multivesicular body, *nu* nucleus, *nucl* nucleolus. For each confocal plain, the fluorescent signal for taxol (TAX) and a differential interference contrast image (DIC) were recorded. The white arrow in **A** indicates a multivesicular body in the process of fusion with the cell membrane

We localised paclitaxel using indirect immunofluorescence with an anti-paclitaxel antibody using a mild fixation protocol with only 1% of paraformaldehyde, which allowed to preserve membrane integrity while allowing penetration of the antibody. In fact, we were able to detect fluorescent signals along the cell wall of glossy cells, but also in the cell interior by collecting confocal z-stacks (Fig. 3A) and the confocal microscopy also allowed to resolve details of the signal. The signal was partially punctate, partially found in larger round clusters of 2–4 µm diameter, where dark, unstained vesicles were seen inside. A comparison of the fluorescent signal with the differential interference contrast image revealed that these signals corresponded to multivesicular bodies close to the plasma membrane. Occasionally, even fusion events of these organelles with the plasma membrane could be caught in action (Fig. 3A, arrow). When we scrutinised confocal sections from deeper in the cell (Fig. 3B), we also detected a fluorescent signal in the spindle-shaped nucleus. Here, the entire karyoplasm was stained, but the signal was excluded from the nucleolus. No signal was detected in the cells during growth phase where the incidence of glossy cells was very limited **(**Suppl. Fig. S3).

To assess, to what extent this signal localisation reflects the situation in living cells (i.e., prior to chemical fixation, which is inevitable for immunofluorescence), we made use of the lipophilic character of paclitaxel that can be stained by Nile Red (Suppl. Fig. S2). Again, we observed a strong fluorescent signal in glossy cells, mostly lining the plasma membrane, but also organised in large vesicular structures that enclosed vesicles of different size and by size and structure matched the structures visualised by paclitaxel immunofluorescence.

### Glossy cells harbour vermiform mitochondria

**Fig. 4:**
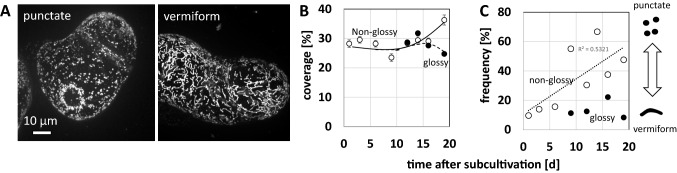
Mitochondrial morphology depends on cell status. **A** Representative cells displaying punctate versus vermiform mitochondria. Geometrical projections of confocal z-stacks collected after labelling with MitoTracker Red. **B** Time course of mitochondrial coverage. Glossy cells could only be scored from day 9, because they were too rare before this time point. **C** Time course of mitochondrial shape change. Data represent the frequency of cells with punctate mitochondria. Low values mean therefore that cells with vermiform mitochondria are abundant. Each data point represents 20–80 individual cells collected from three independent experimental series. Data for non-glossy cells plotted as open circles and for glossy cells as filled circles

Since terminal differentiation is often linked with an oxidative burst in mitochondria (Lam et al. 2001), we wondered, whether there might be changes of mitochondrial morphology in different cell types or at different stages of the cultivation cycle. In fact, when we followed mitochondria by the fluorescent dye Mitotracker Red, we observed that they existed in two forms (Fig. 4A). Either, mitochondria were punctate, or they were vermiform. Within a given cell, the respective mitochondrial morphology was clearly prevailing, e.g., if a cell displayed vermiform mitochondria, it was almost impossible to detect any punctate mitochondrion and vice versa. We quantified mitochondrial coverage over the cell cross-section and followed coverage over the cultivation cycle separately for non-glossy and glossy cells (Fig. 4B). In non-glossy cells, this coverage was constantly at around 30% of the total cross area over almost the entire cultivation cycle, but increased significantly, by around a third from day 15. When, from day 10, glossy cells appeared in significant proportions, these exhibited the same mitochondrial coverage as the non-glossy cells. However, they lacked the terminal increase of coverage seen in their non-glossy companions. While overall coverage did not fluctuate much, there was a clear change in morphology, when the frequency of cells with punctate (i.e., the population without vermiform mitochondria) was scored Fig. 4C. In non-glossy cells, the frequency of cells with punctate mitochondria increased steadily in an approximately linear fashion (upon linear regression, R^2^ was determined as 0.53). The situation was different for glossy cells, that only rarely displayed punctate mitochondria. At the final time point, less than 10% of glossy cells harboured punctate mitochondria, contrasting with the around 50% seen in non-glossy cells. In other words, the predominant fraction of glossy cells produced vermiform mitochondria, representing a clear cytological hallmark for the glossy cell state.

### Conditions that promote glossy cells upregulate specific transcripts of Paclitaxel synthesis

**Fig. 5:**
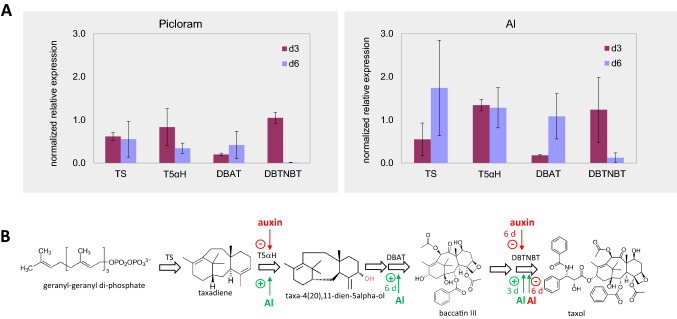
Expression of taxol biosynthesis transcripts. **A** Normalized relative expression of paclitaxel biosynthesis genes after treatment with picloram and aluminium chloride. Data represent mean values from three biological replicates calculated from the DC_t_ values against 18S RNA as internal standard. d3 & d6 indicate the expression values at 3rd and 6th day after the respective treatments. Error bars indicate ± SE. **B** Position of the enzymes encoded by the measured transcripts and effect of auxin (Picloram, 10 µM) versus Al (aluminium chloride, 100 µM). TS taxadiene synthase, T5aH Taxadiene 5a Hydroxylase, DBAT 10-Deacetyl-Baccatin III-10-O-Acetyltransferase, DBTNBT 3‘-N-Debenzoyl-2-Deoxytaxol-N-Benzoyltransferase

To get insight into the metabolic events underlying the increased alkaloid accumulation in response to aluminium ions, we probed the steady-state transcript levels for four key genes of the Paclitaxel biosynthesis pathway by real-time RT-qPCR (Fig. 5) either in response to the synthetic auxin Picloram (which down modulates the incidence of glossy cells), or in response to aluminium ions (which promote the formation of glossy cells). While the transcripts for taxadiene synthase, catalysing the first committed step of the pathway, did not reveal any significant regulation, we observed distinct responses for Taxadiene 5 Hydroxylase (generating the early precursor Taxa-4(20),11-dien-5alpha-ol). Transcripts for this enzyme were significantly lower on treatment with Picloram but induced by aluminium ions. Transcripts for 3 ‘-N-Debenzoyl-2-Deoxytaxol-N-Benzoyltransferase (DBTNBT) were even more responsive. In presence of Picloram, this increase observed at day 3 was completely suppressed at day 6. In response to aluminium ions, there was a late increase of DBAT at day 6 and there was an early increase of DBTNBT transcripts at day 3. In case of DBTNBT, this sharp increase was transient and followed by a sharp decline till day 6. Thus, the regulatory pattern for Taxadiene 5 Hydroxylase, DBAT and for DBTNBT transcripts (suppression by Picloram, stimulation and acceleration in response to aluminium ions) mirrors the pattern seen for the incidence of glossy cells and the accumulation of alkaloids (Fig. 2).

## Discussion

To obtain insight into cellular aspects of metabolic competence in *Taxus* suspension cultures, we discovered a differentiation event that gave rise to a peculiar cell type with a hypertrophic central vacuole of a glossy appearance, when viewed by differential interference contrast. These glossy cells appeared during progressive stages of the cultivation cycle and their frequency could be stimulated by aluminium ions, requiring the activity of NADPH oxidases and actin filaments. The frequency of glossy cells correlated with the accumulation of paclitaxel precursors. When we scrutinised the subcellular details of the glossy cells by immunofluorescence, we could detect paclitaxel in multivesicular bodies that were fusing with the plasma membrane. Similar structures were seen upon live cell imaging using Nile Red. Furthermore, we found that the differentiation into glossy cells coincided with a transition of mitochondria into a vermiform morphology. These findings lead to several questions: What is the link between glossy cell state and alkaloid accumulation? How is this cell state initiated? And, how can it be promoted by aluminium ions? After having discussed these questions, we will show that this phenomenon can be explained as manifestation of the actin–auxin oscillator, a mechanism that calibrates stress signalling against growth in plant cells (for review see Nick 2010), leading to further implications and open questions for future research.

### Cellular “competence” for paclitaxel accumulation 1: role of vacuolar differentiation

The incidence of glossy cells occurs in the later phase of the cultivation cycle (Fig. 2A), and conditions that stimulate the formation of glossy cells are also conditions that stimulate the accumulation of baccatin III and VI (Fig. 2B-D). Thus, the ability of the cells to produce alkaloids correlates with the cell state. Different responses of genetically equal cells to an external factor have been termed as differential “competence” (Mohr 1972). A classical system to study “competence” has been the induction of anthocyanin biosynthesis in the cotyledons of white mustard triggered by activation of the plant photoreceptor phytochrome (Steinitz et al. 1976). This “competence” correlated with the degree of vacuolation (Steinitz and Bergfeld 1977). Using microirradiation, even neighbouring cells in this model system could be shown to be qualitatively different in their response to the light beam, depending on their degree of expansion, whereby “competence” of individual cells did not appear gradually, but in an all-or-none fashion, as result of a developmental switch (Nick et al. 1993). For *Taxus*, as well, the “competence” for the differentiation into a glossy cell (and, hence, for alkaloid accumulation) seems to be linked with vacuolar development. Beginning with day 10 of the cultivation cycle, Packed Cell Volume exhibits a strong step-up accompanied by a sharp step-down in the sugar content of the medium (Suppl. Figure 1C), and a strong increase in fresh weight (Suppl. Figure 1B). These three events can be used as proxy for cell expansion, because dry weight, which correlates with cell number (Suppl. Figure 1B) has approximated saturation at this time point, such that the significant increase in fresh weight seen in this final phase of the cell cycle goes on account of water uptake into the cells. The most straightforward explanation for the concomitant peak in sugar consumption is the massive increase of cell-wall synthesis that is required to balance the rapidly expanding cells against hypo-osmotic burst.

Why should the transition towards vacuolar expansion contribute to the cellular competence for alkaloid accumulation and secretion? When a plant cell accumulates secondary compounds with a toxic effect, it has either to sequester them into the vacuole to safeguard the functionality of the cytoplasm, or it must secrete them out of the cell. Sometimes, both mechanisms even co-exist in different parts of the cell. A classic example is the alkaloid nicotine, which is formed in tobacco roots in response to herbivory, is secreted into the xylem to reach the leaf through the transpiration stream. Once arrived there, it is sequestered in the vacuole, from where it is released upon wounding and, thus, can kill the intruder (for review see Baldwin 2001). A similar phenomenon is seen for the Vinca alkaloids in *Catharanthus roseus* that accumulate in specific idioblasts that, like the glossy cells, are characterised by large vacuoles (for review see Liu et al. 2017) and undergo terminal differentiation into laticifers (Eilert et al. 2011). It is conceivable, therefore, that paclitaxel accumulating cells initiate autolysis, thus, releasing alkaloids into the medium, a mechanism known as lysigenic secretion (for a conceptual review see Turner 1999). There are two arguments speaking against this hypothesis. Lysigenic secretion should go along with an increase of cell mortality. However, stimulation of paclitaxel release into the culture medium was not accompanied by such an increase of mortality (Zhang et al. 2007). If glossy cells would be precursors of lysigenic secretion, the alkaloids should be mainly found in the vacuole. However, neither the immunofluorescence with anti-paclitaxel antibodies (Fig. 3), nor visualisation of lipophilic compounds by Nile Red (Suppl. Fig. S2) detected any signal inside the vacuole, all signals were associated with the plasma membrane or multivesicular bodies in the cytoplasm.

If secretion of paclitaxel is not brought about by lysigenic secretion, it has either to go through membrane transporters, such as the MATE transporters that are responsible for nicotine transport (Shitan et al. 2009), or it is secreted vesicles. There is supporting evidence for both mechanisms. A fluorescent derivative of paclitaxel, flutax, can be taken up into *Taxus* protoplasts (Naill et al. 2012). In protoplasts, the cytoskeleton is disorganised disabling directional vesicle flow, which becomes reinstalled only upon regeneration of a new cell wall (Zaban et al. 2013). Uptake into protoplasts is, therefore, most likely brought about by membrane passage. Since the uptake of flutax is saturable and can be outcompeted with unlabelled paclitaxel, the most straightforward model is that of a specific membrane transporter. In fact, uptake of radioactively labelled paclitaxel into *Taxus* suspension cells was vanadate sensitive (Fornalè et al. 2002), indicative of an ATPase-dependent function, such as it is typical for (ATP-binding cassette) ABC transporters. In fact, a molecular candidate for such a ABC transporter has been identified in *Taxus japonica* (UniProt ID E6Y0T0). However, there is also evidence for secretion. Very recently, the last hydroxylation step in the conversion of baccatin III into the final product paclitaxel, was shown to be brought about by an enzyme that is localised in the endoplasmic reticulum by a transmembrane helix and with a high probability is predicted to act in the secretory pathway (Sanchez-Muñoz et al. 2020). This would also be more compatible with our observation, where both, the paclitaxel signal detected by immunofluorescence (Fig. 3), as well as the Nile Red positive signals (Suppl. Fig. S2) were seen concentrated in secretory organelles, rather than being diffusely distributed across the cytoplasm.

This leads to a different model for paclitaxel secretion that is also able to explain the link between vacuolar differentiation and paclitaxel accumulation in the medium. In recent work by Hanano et al. 2022 on the high producing paclitaxel *Taxus media* cell suspension lines, showed accumulation of paclitaxel in the lipid droplets originating from the ER and the role of caleosins in enhancing paclitaxel biosynthesis. Based on our data obtained on *T. chinesis* suspension cell line, we suggest an alternate model. In our cell lines, the Paclitaxel would initiate its path in the ER, pass the Golgi and reach the multivesicular body. This organelle has two options–in proliferating cells, it can travel to the vacuole and, thus, contributes to the formation of a large central vacuole (for review see Cui et al. 2016). However, in cells that are already fully expanded, the multivesicular body can be re-directed towards the plasma membrane and, thus, contributes to unconventional secretion (for review see Hu et al. 2020). This unconventional secretion is of particular relevance in a defence context (for review see Li et al. 2018), i.e., exactly under the conditions mimicked by addition of MeJA as elicitor.

### Cellular “competence” for paclitaxel accumulation 2: role of mitochondria

The second cytological hallmark of the glossy cell type is the presence of vermiform mitochondria. This mitochondrial shape is often observed in response to hypoxia (van Gestel and Verbelen 2002) and counter intuitively, often comes with oxidative burst in the intermembrane space, because mitochondrial electron transport is perturbed at complex III leading to the accumulation of superoxide as shunt product (reviewed in Wagner et al. 2018). The shift in mitochondrial redox homeostasis is conveyed to the nucleus through retrograde signalling, leading to defence-related cellular responses that depend on the context. A mild stress can be faced, for instance, by activation of mitochondrial superoxide dismutase that will buffer redox homeostasis as concluded from experiments using a mitochondria-targeted peptoid mimick of the ROS scavenger Coenzyme Q (Asfaw et al. 2020). However, in case of a severe stress, as it may be caused by pathogen attack or wounding, the superoxide will not be mitigated, leading to leakage of the inner mitochondrial membrane, the formation of a permeability pore, release of calcium and cytochrome c and, eventually, the activation of programmed cell death (for review see Zancani et al. 2015). Whether hypoxia, expected to occur in cell aggregates, is the trigger for the differentiation into glossy cells, or whether it is a by-product or consequence of vacuolar differentiation, remains to be elucidated. The fact that vermiform mitochondria prevail also in early stages, where glossy cells are not detectable, and only subsequently shift into punctate mitochondria (Fig. 4C) does not support a causative role for differentiation, but rather places the maintenance of vermiform mitochondria as consequence of differentiation.

### Towards a cellular model for paclitaxel “competence”

The differentiation of glossy cells correlates with paclitaxel accumulation, but under control conditions, only around 10% of the cell population display this phenotype (Fig. 2A). This is consistent with a previous report that only a subpopulation of around 5% of the cells accumulate paclitaxel, if not elicited (Naill and Roberts 2005). We observed that MeJA and Al^3+^ ions stimulated both, the incidence of glossy cells, and the accumulation of paclitaxel precursors (Fig. 2B-D). Interestingly, this induction could be suppressed by Diphenylene Iodonium (DPI), an inhibitor of membrane located NADPH oxidases, a central signalling component in plant defence (for review see Marino et al. 2012). This inhibitor of NADPH-dependent flavoproteins inhibits at low concentrations, supporting its specificity. The absence of plant NOS as a second target for DPI, as well as its ability to inhibit at very low concentration to obtain a cellular response emphasize the specificity of DPI against membrane located NADPH oxidases. We, therefore, used DPI to address the role of RboH-dependent oxidative burst. The specific features of this regulatory pattern (induction by MeJA and by aluminium ions, inhibition of this induction by DPI) recapitulate features of a model developed to explain programmed cell death in response to breached membrane integrity (Eggenberger et al. 2017). Here, the NADPH oxidase Respiratory burst oxidase Homologue (RboH) generates superoxide, which can induce the bundling of cortical actin filaments triggering terminal differentiation (Gourlay and Ayscough 2005; Franklin-Tong and Gourlay 2008; Smertenko and Franklin-Tong 2008). This cycle can be mitigated by auxins, which causes debundling of actin by recruiting the superoxide generated by RboH for its own signalling (Eggenberger et al. 2017). An implication of this model is that auxin should be able to mitigate elicitor triggered cell death, because it prevents actin bundling (for review see Nick 2010). This implication has been experimentally confirmed for cell death triggered by harpin, an elicitor from the phytopathogenic bacterium *Erwinia amylovora* (Chang et al. 2015). Our observation that Picloram can suppress the formation of glossy cells (Fig. 2B) indicates that a similar mechanism regulates the cellular competence for paclitaxel biosynthesis. But would such a model of actin remodelling as cellular switch be able to explain the induction by MeJA and by aluminium ions? In fact, MeJA can induce actin bundling (Çetinbaş-Genç and Vardar 2020), and the same holds true for aluminium ions (Ahad and Nick 2007). This aluminium-induced actin bundling can trigger defence genes and is dependent on RboH because the process can be blocked by DPI (Wang et al. 2022). Aluminium can lead to (endogenous) SA, which develops in the context of other defence responses, including actin remodelling (Wang et al. 2022). Exogenous SA, in contrast, hits a cell that had not been primed (in our hands by Al). Thus, the treatment of exogenous SA is not equivalent with that of Al (leading, after some time, to the formation of endogenous SA), because exogenous SA hits a naïve cell, which will, therefore, deploy only a part of the responses compared to a cell, where actin remodelling had already activated defence responses (as seen also be accumulation of paclitaxel biosynthesis transcripts).

**Fig. 6:**
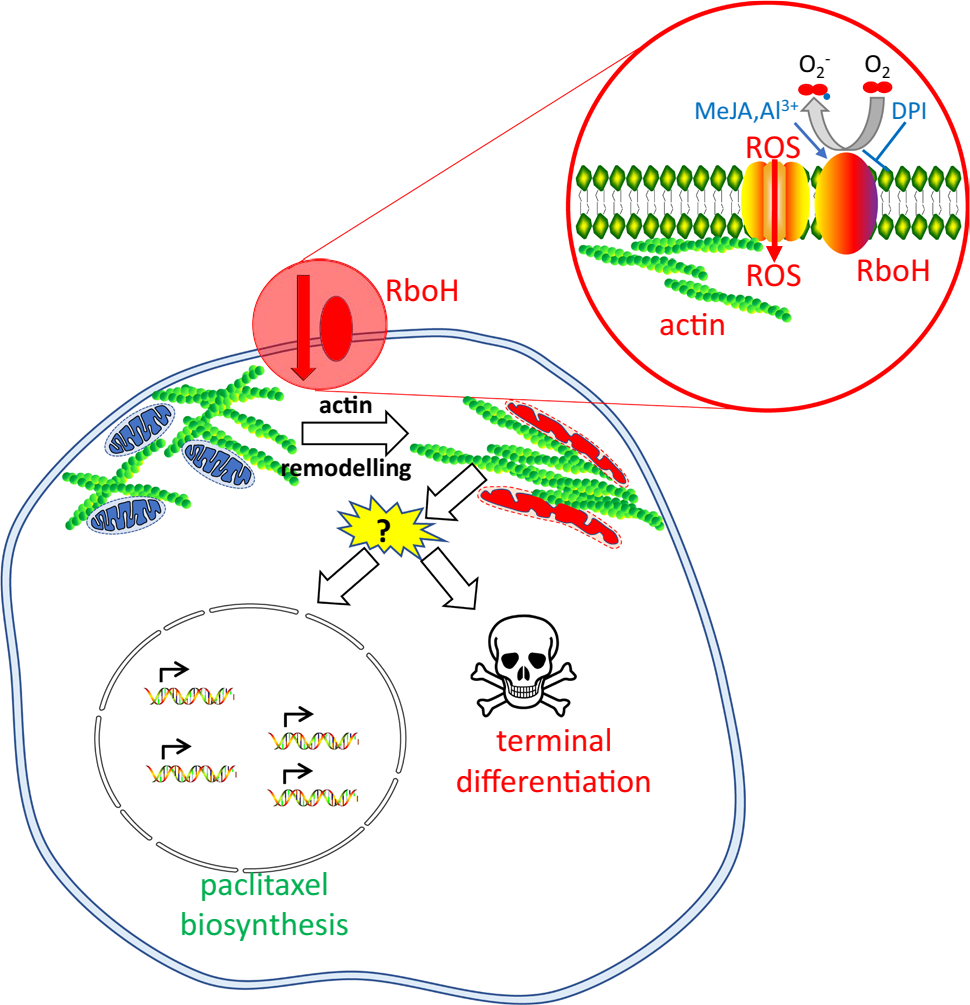
Working model for the role of RboH-dependent actin remodelling for the transition to cellular competence for Paclitaxel metabolism

Thus, the regulatory fingerprint in the induction of cellular competence is matching that of the actin-RboH circuit that is used to sense cellular integrity and by its interaction with the actin–auxin oscillator (Nick 2010; Eggenberger et al. 2017) decides between growth versus terminal differentiation. Applying Occam’s Razor (Clauberg 1691), we arrive at the following working model (Fig. 6) for the suspension cells of *Taxus chinensis* (Pilg.) Rehder used in this study:

Activation of the NADPH oxidase Respiratory burst oxidase Homologue (either in course of culture progression or induced by MeJA or aluminium ions) generates superoxide, which enters the cytoplasm through aquaporins and interferes with the dynamic turnover of actin filaments (for the underlying molecular mechanisms, see Eggenberger et al. 2017). As a result, actin is bundled. Due to the close interaction of actin filaments and plant mitochondria (van Gestel et al. 2002), the remodelling of actin will modulate mitochondrial structure and metabolism, which becomes manifest as the vermiform mitochondria prevalent in glossy cells (Fig. 4). This will then lead to the cellular events commonly associated with mitochondrial perturbation in all eukaryotes (Gourlay and Ayscough 2005), such as formation of a mitochondrial transition pore, leakage of cytochrome C and calcium into the cytoplasm, activation of metacaspases, and eventually programmed cell death. Since actin remodelling also activates defence genes (Wang et al. 2022), the terminal differentiation is also accompanied by induction of biosynthetic genes as precondition for paclitaxel accumulation and secretion.

## Conclusions

Plant cell cultures are not only a promising platform to produce secondary metabolites but can also serve as a system of reduced complexity to address cellular mechanisms. Although plant cells in culture are often considered as “dedifferentiated”, they often preserve certain features from their source tissue (Opatrný et al. 2014). To fully unfold the potential of cell-culture systems, it is important to lend more importance to this innate differentiation between cells. The current work is addressing the link between cellular differentiation and metabolic potency in Paclitaxel accumulation by Taxus suspension cells as paradigm for Plant Cell Fermentation. We can show that the competence for Paclitaxel synthesis correlates with the differentiation of a specific cell type (so called glossy cells) with enlarged and modified vacuoles and a reorganisation of the secretory apparatus. This correlation is not only seen in the temporal development of the two phenomena, but also their regulation in response to elicitors and inhibitors. The competence of these glossy cells is linked with subcellular events, such as the transition of mitochondria from a spherical into a vermiform shape, or the appearance of multivesicular bodies that collect Paclitaxel and secrete it to the medium by fusion with the plasma membrane. It is also accompanied by corresponding induction of transcripts for genes involved in Paclitaxel biosynthesis. The key enzymes seem to be Taxadiene 5 Hydroxylase and DBTNBT. This cellular differentiation can be stimulated by aluminium ions, but inhibited by diphenylene iodonium, a blocker of the membrane located NADP oxidase Respiratory burst oxidase Homologue. Both features indicate that the transition towards the glossy cell state involves actin remodelling. This work highlights the importance of cellular differentiation (even though it is less evident as compared to cells in a tissue context).

### Outlook

The stimulation of glossy cell formation by aluminium ions, the inhibition of this transition by DPI, and the inhibition by exogenous auxin represents a specific hallmark indicating that actin remodelling is steering the transition into the glossy cell state (Fig. 6). A testable implication of our hypothesis would be that Latrunculin B, a specific compound sequestering G-actin from assembly into F-actin, should suppress the frequency of glossy cells as well. Furthermore, generation of a fluorescent actin-marker line expressing the actin-binding domain of plant fimbrin in fusion with GFP would allow to visualise the aluminium induced remodelling directly (Wang et al. 2022). For the context of Plant Cell Fermentation, it might be rewarding to focus initially on sustaining cell proliferation which leads to accumulation of biomass exclusively. Once this has been achieved, triggers could be then set to allow differentiation into glossy cells which may lead to activation of metabolic potency to reach higher product yields. Another interesting research avenue would be to understand epigenetic changes if any between the non-glossy and glossy cell types. As a new emerging field (Brzycki et al. 2021), to look for epigenetic changes would be certainly rewarding, here, one might test the effect of compounds that interfere with histone acetylation. Thus, understanding epigenetic regulation on these Taxus cell lines could perhaps identify epigenetic engineering targets to produce more glossy cells for increased paclitaxel production.

## Supplementary Information

Below is the link to the electronic supplementary material.

Supplementary file1 (PPTX 59 KB)

Supplementary file2 (PPTX 402 KB)

Supplementary file3 (PPTX 2803 KB)

Supplementary file4 (DOCX 14 KB)

## Data Availability

The datasets presented in this study are stored on the server of the Steinbuch Centre for Computing and are made available on reasonable request.

## References

[CR1] Abe M, Shibaoka H, Yamane H, Takahashi N (1990). Cell cycle-dependent disruption of microtubules by methyl jasmonate in tobacco BY-2 cells. Protoplasma.

[CR2] Ahad A, Nick P (2007). Actin-bundling in activation-tagged aluminum-tolerant tobacco mutants. Planta.

[CR3] Altmann KH, Gertsch J (2007). Anticancer drugs from nature—natural products as a unique source of new microtubule-stabilizing agents. Nat Prod Rep.

[CR4] Asfaw KG, Eghbalian R, Liu Q, Maisch J, Münch S, Wehl I, Bräse S, Bogeski I, Schepers U, Nick P (2020). A mitochondria-targeted coenzyme q peptoid induces superoxide dismutase and alleviates salinity stress in plant cells. Nat Sci Rep.

[CR5] Baldwin IT (2001). An ecologically motivated analysis of plant-herbivore interactions in native tobacco. Plant Physiol.

[CR6] Baloglu E, Kingston DGI (1999). The taxane diterpenoids. J Nat Prod.

[CR7] Besumbes O, Sauret-Güeto S, Phillips MA, Imperial S, Rodríguez-Concepción M, Boronat A (2004). Metabolic engineering of isoprenoid biosynthesis in Arabidopsis for the production of taxadiene, the first committed precursor of Taxol. Biotechnol Bioeng.

[CR8] Boller T, Felix G (2009). A renaissance of elicitors: perception of microbe-associated molecular patterns and danger signals by pattern-recognition receptors. Annu Rev Plant Biol.

[CR9] Bringi V, Kadkade P, Prince C, Roach B (2012) Enhanced production of Paclitaxel and taxanes by cell culturs of a Taxus 672 species. US patent USOO8338143B2. https://patents.google.com/patent/US8338143B2/en. Accessed 6 Oct 2022

[CR10] Brzycki CM, Young EM, Roberts SC, Malik S (2021). Secondary metabolite production in plant cell culture: A new epigenetic frontier. Exploring plant cells for the production of compounds of interest.

[CR11] Çetinbaş-Genç A, Vardar F (2020). Effect of methyl jasmonate on in-vitro pollen germination and tube elongation of *Pinus nigra*. Protoplasma.

[CR12] Chang X, Riemann M, Nick P (2015). Actin as deathly switch? How auxin can suppress cell-death related defence. PLoS ONE.

[CR13] Clauberg J, Schalbruch JT (1691) Opera omnia philosophica. Blaeu, Amsterdam

[CR14] Covello PS (2008). Making artemisinin. Phytochemistry.

[CR15] Cui Y, Shen J, Gao C, Zhuang X, Wang J, Jiang L (2016). Biogenesis of Plant Prevacuolar Multivesicular Bodies. Mol Plant.

[CR16] Cusido RM, Onrubia M, Sabater-Jara AB, Moyano E, Bonfill M, Goossens A, Angeles Pedreño M, Palazon J (2014). A rational approach to improving the biotechnological production of taxanes in plant cell cultures of *Taxus spp*. Biotechnol Adv.

[CR17] Duan D, Fischer S, Merz P, Bogs J, Riemann M, Nick P (2016). An ancestral allele of grapevine transcription factor MYB14 promotes plant defence. J Exp Bot.

[CR18] Eggenberger K, Sanyal P, Hundt S, Wadhwani P, Ulrich AS, Nick P (2017). Challenge Integrity: The Cell-Penetrating Peptide BP100 Interferes with the Auxin–Actin Oscillator.. Plant Cell Physiol.

[CR19] Eilert U, Nesbitt LR, Constabel F (2011). Laticifers and latex in fruits of periwinkle, *Catharanthus roseus*. Can J Bot.

[CR20] Farjon A, Page CN(1999). Conifers: Status survey and conservation action plan. Gland: IUCN. https://portals.iucn.org/library/node/7565. Accessed 14 April 2022

[CR21] Farmer EE, Ryan CA (1990). Interplant communication: airborne methyl jasmonate induces synthesis of proteinase inhibitors in plant leaves. Proc Natl Acad Sci USA.

[CR22] Fornalè S, Esposti DD, Navia-Osorio A, Cusidò RM, Palazòn J, Piñol MT, Bagni N (2002). Taxol transport in *Taxus baccata* cell suspension cultures. Plant Physiol Biochem.

[CR23] Franklin-Tong VE, Gourlay CW (2008). A role for actin in regulating apoptosis/programmed cell death: evidence spanning yeast, plants and animals. Biochem J.

[CR24] Gómez-Galera S, Pelacho AM, Gené A, Capell T, Christou P (2007). The genetic manipulation of medicinal and aromatic plants. Plant Cell Rep.

[CR25] Goossens A, Häkkinen ST, Laakso I, Seppänen-Laakso T, Biondi S, De Sutter V, Lammertyn F, Nuutila AM, Söderlund H, Zabeau M, Inzé D, Oksman-Caldentey KM. (2003) A functional genomics approach toward the understanding of secondary metabolism in plant cells.Proc Natl Acad Sci USA. 100(14): 8595–8600. 10.1073/pnas.1032967100 PMC166274 12826618

[CR26] Gourlay CW, Ayscough KR (2005). The actin cytoskeleton: a key regulator of apoptosis and ageing?. Nat Rev Mol Cell Biol.

[CR27] Guan X, Buchholz G, Nick P (2015). Tubulin marker line of grapevine suspension cells as a tool to follow early stress responses. J Plant Phys.

[CR28] Hanano A, Perez-Matas E, Shaban M (2022). Characterization of lipid droplets from a *Taxus media* cell suspension and their potential involvement in trafficking and secretion of paclitaxel. Plant Cell Rep.

[CR29] Hu S, Li Y, Shen J (2020). A diverse membrane interaction network for plant multivesicular bodies: roles in proteins vacuolar delivery and unconventional secretion. Front Plant Sci.

[CR30] Jordan M, Wilson L (2004). Microtubules as a target for anticancer drugs. Nat Rev Cancer.

[CR31] Jovanovic A, Durst S, Nick P (2010). Plant cell division is specifically affected by nitrotyrosine. J Exp Bot.

[CR32] Kingston DG, Chordia MD, Jagtap PG, Liang J, Shen YC, Long BH, Fairchild CR, Johnston KA (1999). Synthesis and biological evaluation of 1-Deoxypaclitaxel analogues. J Org Chem.

[CR33] Kössel A (1891) Über die chemische Zusammensetzung der Zelle. Du Bois-Reymond’s Archiv/Arch Anat Physiol Physiol Abt, Veit & Comp, Leipzig. pp 181–186.

[CR34] Kovacs K, Zhang L, Linforth RS, Whittaker B, Hayes CJ, Fray RG (2007). Redirection of carotenoid metabolism for the efficient production of taxadiene [taxa-4(5),11(12)-diene] in transgenic tomato fruit. Transgenic Res.

[CR35] Küpeli E, Erdemoglu N, Yesilada E, Sener B (2003). Anti-inflammatory and antinociceptive activity of taxoids and lignans from the heartwood of Taxus baccata L. J Ethnopharmacol.

[CR36] Lam E, Kato N, Lawton M (2001). Programmed cell death, mitochondria and the plant hypersensitive response. Nature.

[CR37] Laskaris G, Bounkhay M, Theodoridis G, van der Heijden R, Verpoorte R, Jaziri M (1999). Induction of geranylgeranyl diphosphate synthase activity and taxane accumulation in *Taxus baccata* cell cultures after elicitation by methyl jasmonate. Plant Sci.

[CR38] Lee EK, Jin YW, Park JH, Yoo YM, Hong SM, Amir R, Yan Z, Kwon E, Elfick A, Tomlinson S, Halbritter F, Waibel T, Yun BW, Loake GJ (2010). Cultured cambial meristematic cells as a source of plant natural products. Nature Biotechnol.

[CR39] Li X, Bao H, Wang Z, Wang M, Fan B, Zhu C, Chen Z (2018). Biogenesis and function of multivesicular bodies in plant immunity. Front Plant Sci.

[CR40] Lin SL, Wei T, Lin JF, Guo LQ, Wu GP, Wei JB, Huang JJ, Ouyang PL (2018). Bio-production of baccatin iii, an important precursor of paclitaxel by a cost-effective approach. Mol Biotechnol.

[CR41] Liu J, Cai J, Wang R, Yang S (2017). Transcriptional regulation and transport of terpenoid indole alkaloid in catharanthus roseus: exploration of new research directions. Int J Mol Sci.

[CR42] Livak KJ, Schmittgen TD (2001). Analysis of relative gene expression data using real-time quantitative PCR and the 2^− ΔΔCT^ method. Methods.

[CR43] Maisch J, Nick P (2007). Actin is involved in auxin-dependent patterning. Plant Physiol.

[CR44] Malik S, Cusidó RM, Mirjalili MH, Moyano E, Palazón J, Bonfill M (2011). Production of the anticancer drug taxol in Taxus baccata suspension cultures: a review. Process Biochem.

[CR45] Marino D, Dunand C, Puppo A, Pauly N (2012). A burst of plant NADPH oxidases. Trends Plant Sci.

[CR46] Mohr H (1972). Light-mediated Flavonoid Synthesis: A Biochemical Model System of Differentiation. Lectures on Photomorphogenesis.

[CR47] Monteiro LB, Davanzo GG, de Aguiar CF, Moraes-Vieira PMM (2020). Using flow cytometry for mitochondrial assays. Methods X.

[CR48] Morimoto S, Suemori K, Moriwaki J, Taura F, Tanaka H, Aso M, Tanaka M, Suemune H, Shimohigashi Y, Shoyama Y (2001). Morphine metabolism in the opium poppy and its possible physiological function. J Biol Chem.

[CR49] Naill MC, Roberts SC (2005). Flow cytometric identification of Paclitaxel-accumulating subpopulations. Biotechnol Prog.

[CR50] Naill MC, Kolewe ME, Roberts SC (2012). Paclitaxel uptake and transport in *Taxus* cell suspension cultures. Flutax uptake into Taxus protoplast, saturable and competing with cold paclitaxel, indicating a membrane transporter. Biochem Eng J.

[CR51] Nasiri J, Naghavi MR, Alizadeh H, Moghadam MR (2016). Seasonal-based temporal changes fluctuate expression patterns of TXS, DBAT, BAPT and DBTNBT genes alongside production of associated taxanes in *Taxus baccata*. Plant Cell Rep.

[CR52] Newman DJ, Cragg GM, Snader KM (2003). Natural products as sources of new drugs over the period 1981–2002. J Nat Prod.

[CR53] Nick P (2010). Probing the actin-auxin oscillator. Plant Signaling Behav.

[CR54] Nick P, Ehmann B, Furuya M, Schäfer E (1993). Cell communication, stochastic cell responses, and anthocyanin pattern in mustard cotyledons. Plant Cell.

[CR55] Nick P, Heuing A, Ehmann B (2000). Plant chaperonins: a role in microtubule-dependent wall-formation?. Protoplasma.

[CR56] Nicolaou KC, Yang Z, Liu JJ, Ueno H, Nantermet PG, Guy RK, Claiborne CF, Renaud J, Couladouros EA, Paulvannan K (1994). Total synthesis of taxol. Nature.

[CR57] Nims E, Dubois CP, Roberts SC, Walker EL (2006). Expression profiling of genes involved in paclitaxel biosynthesis for targeted metabolic engineering. Metabolic Engineer.

[CR58] Onrubia M, Moyano E, Bonfill M, Expósito O, Palazón J, Cusidó RM (2010). An approach to the molecular mechanism of methyl jasmonate and vanadyl sulphate elicitation in *Taxus baccata* cell cultures: The role of *txs* and *bapt* gene expression. Biochem Engineer J.

[CR59] Opatrný Z, Nick P, Petrášek J (2014). Plant cell strains in fundamental research and applications. Plant Cell Monogr.

[CR60] Rajabi F, Heene E, Maisch J, Nick P (2017). Combination of plant metabolic modules yields synthetic synergies. PLoS ONE.

[CR61] Ramirez-Estrada K, Osuna L, Moyano E, Bonfill M, Tapia N, Cusido RM, Palazon J (2015). Changes in gene transcription and taxane production in elicited cell cultures of *Taxus × media* and *Taxus globosa*. Phytochemistry.

[CR62] Ramirez-Estrada K, Vidal-Limon H, Hidalgo D, Moyano E, Golenioswki M, Cusidó RM, Palazon J (2016). Elicitation, an effective strategy for the biotechnological production of bioactive high-added value compounds in plant cell factories. Molecules.

[CR01] Rao SR, Ravishankar GA (2002). Plant cell cultures: Chemical factories of secondary metabolites. Biotechnol Adv.

[CR63] Sanchez-Muñoz R, Perez-Mata E, Almagro L, Cusido R, Bonfill M, Palazon J, Moyano E (2020). A novel Hydroxylation step in the taxane biosynthetic pathway: a new approach to paclitaxel production by synthetic biology. Front Bioeng Biotechnol.

[CR64] Shitan N, Morita M, Kazufumi Yazaki K (2009). Identification of a nicotine transporter in leaf vacuoles of *Nicotiana tabacum*. Plant Signal Behav.

[CR65] Shoji T, Hashimoto T (2008). Why does anatabine, But not nicotine, accumulate in jasmonate-elicited cultured tobacco BY-2 cells?. Plant Cell Physiol.

[CR66] Smertenko A, Franklin-Tong VE (2008). Organisation and regulation of the cytoskeleton in plant programmed cell death. Cell Death Differ.

[CR67] Steinitz B, Bergfeld R (1977). Pattern formation underlying phytochrome-mediated anthocyanin synthesis in the cotyledons of *Sinapis alba* L. Planta.

[CR68] Steinitz B, Drumm H, Mohr H (1976). The appearance of competence for phytochrome-mediated anthocyanin synthesis in the cotyledons of *Sinapis alba* L. Planta.

[CR69] Svyatyna K, Jikumaru Y, Brendel R, Reichelt M, Mithöfer A, Takano M, Kamiya Y, Nick P, Riemann M (2014). Light induces jasmonate-isoleucine conjugation via OsJAR1-dependent and -independent pathways in rice. Plant Cell Environment.

[CR70] Thornburg CK, Walter T, Walker KD (2017). Biocatalysis of a paclitaxel analogue: conversion of Baccatin III to N-Debenzoyl-N-(2-furoyl) paclitaxel and characterization of an Amino Phenylpropanoyl CoA transferase. Biochemistry.

[CR71] Turner GW (1999). A brief history of the lysigenous gland hypothesis. Bot Rev.

[CR72] van der Heijden R, Jacobs DI, Snoeijer W, Hallard D, Verpoorte R (2004). The Catharanthus alkaloids: pharmacognosy and biotechnology. Curr Med Chem.

[CR73] van Gestel K, Köhler RH, Verbelen JP (2002). Plant mitochondria move on F-actin, but their positioning in the cortical cytoplasm depends on both F-actin and microtubules. J Exp Bot.

[CR74] van Gestel K, Verbelen JP (2002). Giant mitochondria are a response to low oxygen pressure in cells of tobacco (*Nicotiana tabacum* L.). J Exp Bot.

[CR75] Verpoorte R (1998). Exploration of nature's chemodiversity: the role of secondary metabolites as leads in drug development. Drug Discov Today.

[CR76] Vidensek N, Lim P, Campbell A, Carlson C (1990). Taxol content in bark, wood, root, leaf, twig, and seedling from several *Taxus* species. J Nat Prod.

[CR77] Wagner S, Aken OV, Elsässer M, Schwarzländer M (2018). Mitochondrial energy signaling and its role in the low-oxygen stress response of plants. Plant Physiol.

[CR78] Wang H, Riemann M, Liu Q, Siegrist J, Nick P (2021). Glycyrrhizin, the active compound of the TCM drug Gan Cao stimulates actin remodelling and defence in grapevine. Plant Sci.

[CR79] Wang R, Dong D, Metzger C, Zhu X, Riemann M, Pla M, Nick P (2022). Aluminium can activate grapevine defence through actin remodelling. Horticultural Res.

[CR80] Widholm JM (1972). The use of fluorescein diacetate and phenosafranine for determining viability of cultured plant cells. Stain Technol.

[CR81] Witherup KM, Look S, Stasko M, McCloud T, Issaq H, Muschik G (1989). High performance liquid chromatographic separation of taxol and related compounds from *Taxus brevifolia*. J Liquid Chrom Related Technol.

[CR82] Yue W, Ming QL, Lin B, Rahman K, Zheng CJ, Han T, Qin LP (2002). Medicinal plant cell suspension cultures: pharmaceutical applications and high-yielding strategies for the desired secondary metabolites. Crit Rev Biotechnol.

[CR02] Yue W, Ming QL, Lin B, Rahman K, Zheng CJ, Han T, Qin LP (2016). Medicinal plant cell suspension cultures:pharmaceutical applications and high-yielding strategies for the desired secondary metabolites. Crit RevBiotechnol.

[CR83] Zaban B, Maisch J, Nick P (2013). Dynamic actin controls polarity induction de novo in protoplasts. J Int Plant Biol.

[CR84] Zancani M, Casolo V, Petrussa E, Peresson C, Patui S, Bertolini A, De Col V, Braidot E, Boscutti F, Vianello A (2015). The Permeability Transition in plant mitochondria: the missing link. Front Plant Sci.

[CR85] Zhang ChH, Fevereiro PS, He GY, Chen ZhJ (2007). Enhanced paclitaxel productivity and release capacity of *Taxus chinensis* cell suspension cultures adapted to chitosan. Plant Sci.

