# Supplementary material for: Switching cell fate by the actin–auxin oscillator in Taxus: cellular aspects of plant cell fermentation
Source: Plant Cell Rep. 2022 Oct 10;41(12):2363–78. doi: 10.1007/s00299-022-02928-0 (PMC9700576; doi:10.1007/s00299-022-02928-0)
Supplement: Supplementary file 1 — Supplementary file1 (PPTX 59 KB) [file 299_2022_2928_MOESM1_ESM.pptx]

## Slide 1
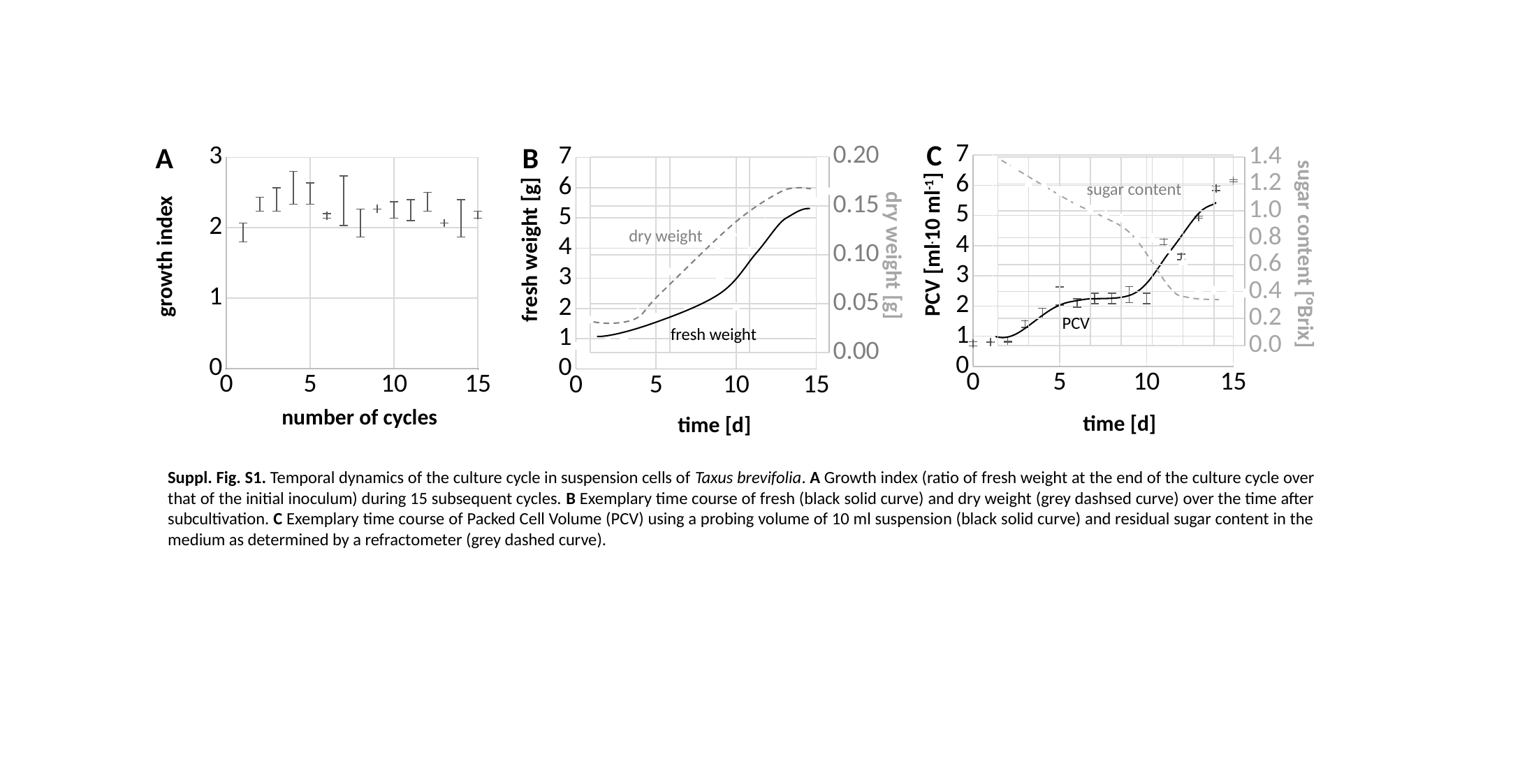

C
B
A
### Chart
| Category | |
|---|---|
### Chart
| Category | |
|---|---|
### Chart
| Category | mean |
|---|---|
### Chart
| Category | |
|---|---|
### Chart
| Category | dw |
|---|---|
sugar content
dry weight
PCV [ml.10 ml-1]
fresh weight [g]
sugar content [°Brix]
dry weight [g]
growth index
PCV
fresh weight
number of cycles
time [d]
time [d]
Suppl. Fig. S1. Temporal dynamics of the culture cycle in suspension cells of Taxus brevifolia. A Growth index (ratio of fresh weight at the end of the culture cycle over that of the initial inoculum) during 15 subsequent cycles. B Exemplary time course of fresh (black solid curve) and dry weight (grey dashsed curve) over the time after subcultivation. C Exemplary time course of Packed Cell Volume (PCV) using a probing volume of 10 ml suspension (black solid curve) and residual sugar content in the medium as determined by a refractometer (grey dashed curve).
