# Supplementary material for: Switching cell fate by the actin–auxin oscillator in Taxus: cellular aspects of plant cell fermentation
Source: Plant Cell Rep. 2022 Oct 10;41(12):2363–78. doi: 10.1007/s00299-022-02928-0 (PMC9700576; doi:10.1007/s00299-022-02928-0)
Supplement: Supplementary file 2 — Supplementary file2 (PPTX 402 KB) [file 299_2022_2928_MOESM2_ESM.pptx]

## Slide 1
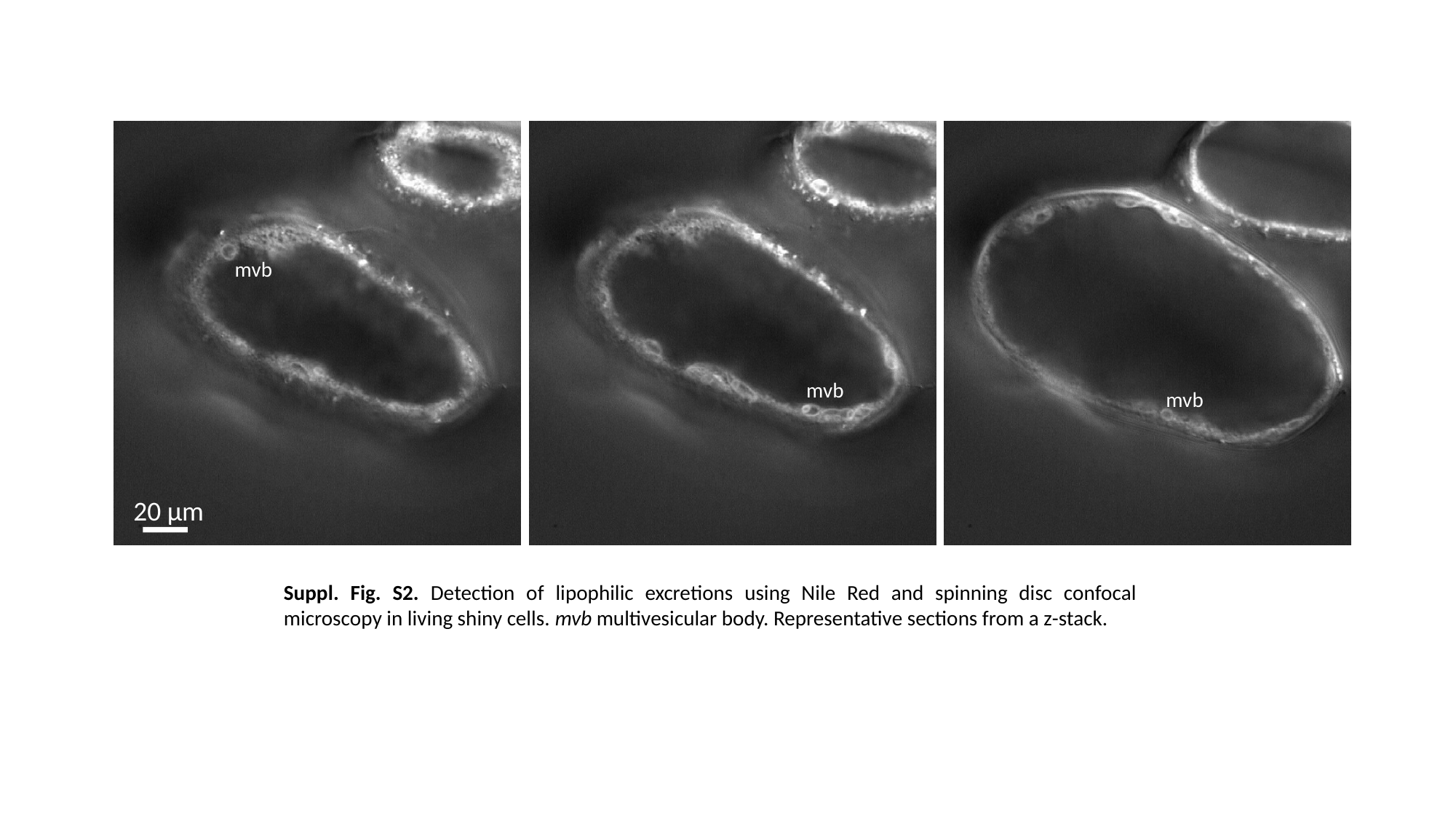

mvb
mvb
mvb
20 µm
Suppl. Fig. S2. Detection of lipophilic excretions using Nile Red and spinning disc confocal microscopy in living shiny cells. mvb multivesicular body. Representative sections from a z-stack.
