# Supplementary material for: Switching cell fate by the actin–auxin oscillator in Taxus: cellular aspects of plant cell fermentation
Source: Plant Cell Rep. 2022 Oct 10;41(12):2363–78. doi: 10.1007/s00299-022-02928-0 (PMC9700576; doi:10.1007/s00299-022-02928-0)
Supplement: Supplementary file 3 — Supplementary file3 (PPTX 2803 KB) [file 299_2022_2928_MOESM3_ESM.pptx]

## Slide 1
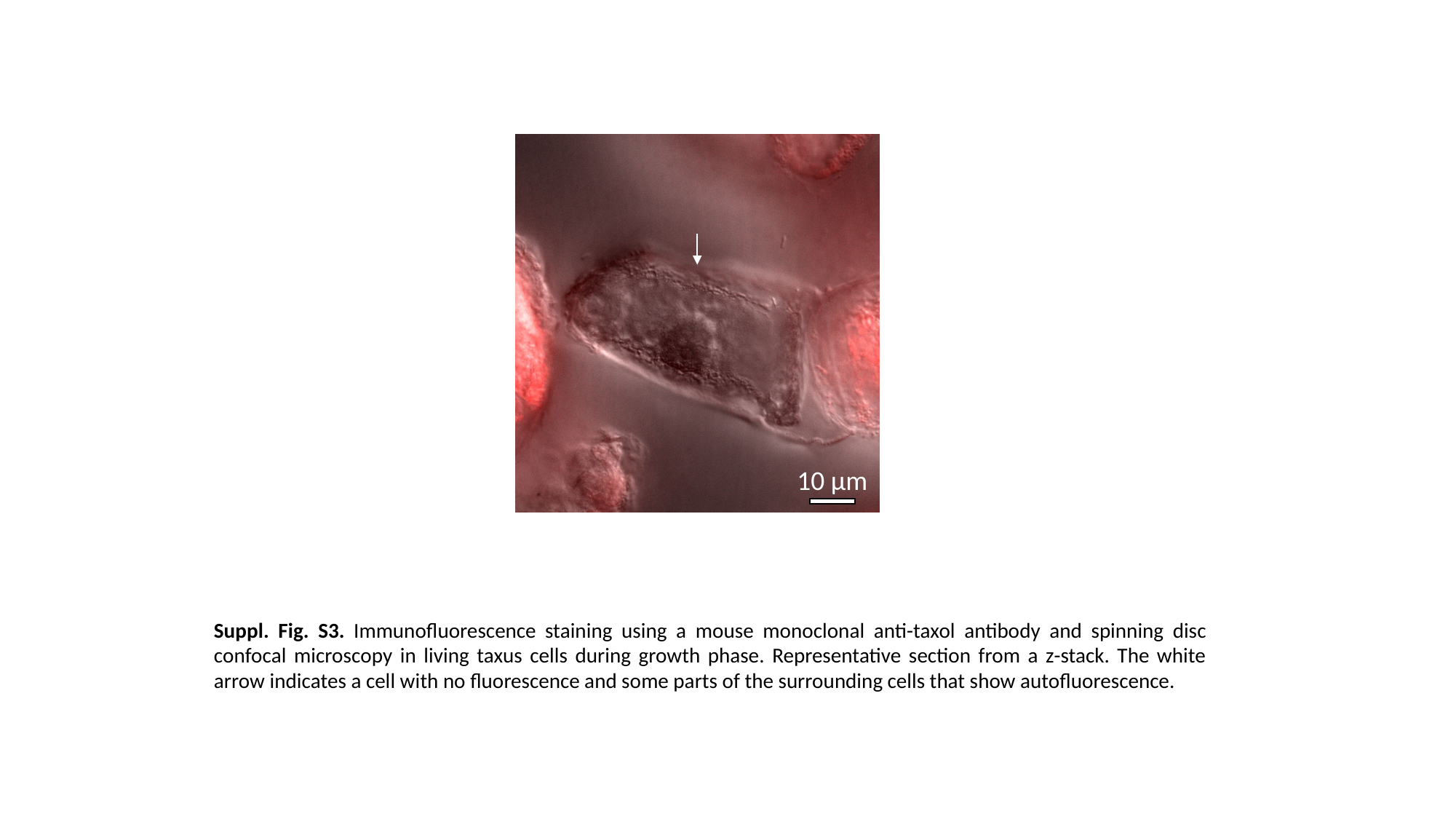

10 µm
Suppl. Fig. S3. Immunofluorescence staining using a mouse monoclonal anti-taxol antibody and spinning disc confocal microscopy in living taxus cells during growth phase. Representative section from a z-stack. The white arrow indicates a cell with no fluorescence and some parts of the surrounding cells that show autofluorescence.
