# Supplementary material for: Switching cell fate by the actin–auxin oscillator in Taxus: cellular aspects of plant cell fermentation
Source: Plant Cell Rep. 2022 Oct 10;41(12):2363–78. doi: 10.1007/s00299-022-02928-0 (PMC9700576; doi:10.1007/s00299-022-02928-0)
Supplement: Supplementary file 4 — Supplementary file4 (DOCX 14 KB) [file 299_2022_2928_MOESM4_ESM.docx]

**Supplementary Table S1.** Oligonucleotide primers to measure transcripts of Paclitaxel biosynthesis by real-time qPCR. TS Taxadiene Synthase, T5αH Taxadiene 5α Hydroxylase, DBAT 10-Deacetyl-Baccatin III-10-O-Acetyltransferase, DBTNBT 3‘-N-Debenzoyl-2-Deoxytaxol-N-Benzoyltransferase

| target | primer sequence | T_m_ (°C) | reference |
| --- | --- | --- | --- |
| 18S rRNA | 5’-CCGCGGTAATTCCGCTCCAAT-3’  5’-GAGGGCCAGTGCACACCGAGTA-3’ | 58 | Onrubia et al. (2010) |
| TS | 5’-AATGCAGCGCTGAAGATGAATGCA-3’  5’-TTGGCTCTGCCCTGTTTTCCAAC-3’ | 58 | Nims et al. (2006) |
| T5αH | 5’-TTAGGCATCCCTTTCATTGG-3’  5’-ACATCTGCACCAGCTTCTCC-3’ | 56 | Nims et al. (2006) |
| DBAT | 5’-GGGATCTTGAAGTGGAGTGC-3’  5’-ACCATGGCAGAAACTCATCC-3’ | 58 | Nims et al. (2006) |
| DBTNBT | 5’-GCAGGGGAATTTTTAACACG-3’  5’-ATGGCTTCCACAAACAGAGC-3’ | 52 | Nims et al. (2006) |
